# Lack of identification of *Flaviviruses* in oral and cloacal swabs from long- and short-distance migratory birds in Trentino-Alto Adige (North-eastern Italy)

**DOI:** 10.1186/1743-422X-10-306

**Published:** 2013-10-11

**Authors:** Michela Grisenti, Daniele Arnoldi, Franco Rizzolli, Mario Giacobini, Luigi Bertolotti, Annapaola Rizzoli

**Affiliations:** 1 Edmund Mach Foundation, via E. Mach 1, San Michele all’Adige, Trento, Italy; 2 Department of Veterinary Sciences, University of Torino, via L. Da Vinci 44, Grugliasco, Torino, Italy

**Keywords:** Flavivirus, Migratory birds, Oral and cloacal swabs, Survey

## Abstract

**Background:**

West Nile virus (WNV) and Usutu virus (USUV), both belonging to the genus *Flavivirus*, are emerging in Italy as important human and animal pathogens. Migratory birds are involved in the spread of *Flaviviruses* over long distances, particularly from Africa to Europe. Once introduced, these viruses can be further be dispersed by short-distance migratory and resident bird species. Thus far, there is still a considerable knowledge gap on the role played by different bird species in the ecology and transmission mechanisms of these viruses. The Region of Trentino-Alto Adige (north-eastern Italy) is located on the migratory route of many of the short- and long-distance migratory birds that cross the Alps, connecting northern Europe and western Asia with southern Europe and Africa. Until now, only a silent circulation of WNV and USUV within the territory of the Province of Trento has been confirmed by serological screening, whilst no cases of infected humans or animals have so far been reported. However, continuous spillover events of both viruses have been reported in neighbouring Regions. The aim of this study was to monitor the circulation of WNV and USUV in Trentino-Alto Adige, in order to detect if active virus shedding occurs in migratory birds captured during their seasonal movements and to evaluate the role that different bird species could play in the spreading of these viruses.

**Methods:**

We carried out a biomolecular survey on oral and cloacal swabs collected from migratory birds during seasonal migrations. Birds belonging to 18 transaharian and 21 intrapaleartic species were examined during spring (n = 176) and autumn (n = 146), and were tested using a generic nested-PCR.

**Results:**

All samples tested negative for Flaviviruses. The possible causes of unapparent shedding, along with ecological and epidemiological implications are discussed.

**Conclusions:**

The lack of detection of active virus shedding in these bird species does not exclude the circulation of these viruses within the Trentino-Alto Adige region, as reported in previous studies. The possible ecological implications are discussed.

## Background

The genus *Flavivirus*, family *Flaviviridae*, contains more than 70 viruses subdivided into three groups, according to their route of transmission: 1) arthropod-borne, infecting a range of vertebrate hosts through mosquito or tick bites; 2) those spread by an unknown vector, presumed to be limited to infecting vertebrates only, or; 3) those spread by insects only, called ‘insect-specific flaviviruses’ or ‘mosquitoes-only flaviviruses’, because they replicate only in mosquito-derived cells. When considering their observed pathogenicity for humans, those with highest impact on human health in Europe belong to the first group, and include West Nile Virus (WNV), Usutu Virus (USUV), and Tick-borne Encephalitis Virus [1,2] and references therein.

WNV is a zoonotic agent that has been reported in Africa since the beginning of the 20^th^ century and has since then radiated into Europe, India, Asia, Australia and America. During the last decade, new strains with various pathogenic characteristics have been discovered [3-5]. WNV is maintained in nature by a cycle involving ornithophilic mosquitoes as the vector, principally *Culex* spp., and birds that are the amplifying hosts. It infects a broad range of avian and mammalian species, but has also been reported to infect reptiles and amphibians. Other mechanisms of transmission include mites and ticks, organ transplant, blood transfusion, breastfeeding, intrauterine infection, and the fecal-oral route [6-11] and references therein. In Italy, WNV lineage 1 has been circulating since 1998 [12]. Surveillance activities established in 15 Italian wetlands from 2001 to 2007 detected only sporadic WNV circulations in several areas through seroconversions in chickens and horses [13-15] and references therein. Since 2008, WNV lineage 1 has been detected in animals, mosquitoes, and humans in an increasing number of Italian Regions each year, with clinical symptoms reported in horses and humans [16]. In 2011, the first human infection of WNV lineage 2 was discovered in central Italy, and later detected in birds and mosquitoes in north-eastern Italy and Sardinia [17,18]. Based on phylogenetic analyses, isolated strains were grouped in eight distinct lineages [19]. WNV infection results in flu-like symptoms or neurological disorders with heavy sequelae and eventually death. Many studies, however, have shown that this virus can circulate silently, infecting animals and humans asymptomatically [5,8,14,20,21].

USUV is another *Flavivirus* isolated for the first time from *Culex neavei* (*Cx. neavei*) mosquitoes in South Africa in 1959. It is maintained in nature by a mosquito-bird transmission cycle, with the genus *Culex* as the main vector, and for several years it has been considered a virus with very low pathogenicity for humans and animals [22]. It was historically only detected in tropical and subtropical Africa. However, the first European cases were confirmed in Italy in 1996 [23] and then in Austria in 2001 [24], resulting in the deaths of several species of resident birds, including Blackbirds (*Turdus merula*), Great Gray Owls (*Strix nebulosa*) and Barn Swallows (*Hirundo rustica*) [23,24]. In the following years, the virus was detected in birds and/or mosquitoes of several countries, including more cases in Italy [14,25], Switzerland [26], the UK [27] and Germany [28]. Moreover, the virus appears to have increased in pathogenicity, with fatalities in European wild birds [23,26]. In 2009, the virus was linked to neurological disorders in humans for the first time in Italy [25] and references therein.

Wild birds are believed to have the potential to maintain, transport, and disperse several *Flaviviruses*, as reviewed by some authors e.g.: [29]. Wild birds living in Africa, Europe and Asia can be divided in migratory and non-migratory (or ‘resident’). The latter permanently live in the territory where they are born and travel only short distances to search for food and new ecosystems. Migratory birds annually undertake journeys, principally in spring and autumn, from their reproductive territory to where they will spend the winter (overwintering grounds) and viceversa. The former include intrapaleartic (or short-distance) migrants moving between Europe, Asia and North Africa; whilst others are long-distance or transaharian migrants, flying between Europe and southern Africa. Since the first appearance of WNV in North America in 1999 [30], much research has been carried out to understand the epidemiological role of bird species, demonstrating that migratory birds are implicated in the spread of diseases over long distances, such as from Africa into Europe, while the successive spread at a local level is mainly induced by resident and short-distance migrants, both for WNV and USUV e.g.: [23,26,29,31] and references therein. At the stopover sites along their migratory route and once they reach their destination grounds, migratory birds share common habitats with resident species from which they are otherwise separated during the rest of the year, and this exposes them to a great range of vectors and pathogens. The physiological stress of migration can increase their susceptibility to WNV, and/or lead to the reactivation of latent and chronic infections [29,32,33] and references therein.

Among the non-vectorial transmission routes of WNV between birds, oral and fecal viral shedding plays a central epidemiological role for many reasons. The fecal-oral secretions and excretions can contaminate the environment, leading to a high number of individuals coming into contact with the virus. In addition, this transmission route can take place in several ways, such as direct and indirect contact (e.g.: inhalation of aerosols, ingestion of contaminated food, preening soiled feathers), intra- and inter-species socialization, feeding of the nestlings, cannibalism and scavenging of infected carcasses. In fact, the viremia in orally-infected animals is similar to the one reached after mosquito bites [7,8,10,11,34] and references therein. Furthermore, oral and fecal shedding may last longer than the viraemic phase (usually less than 7 days [11]), can occur without apparent clinical signs, and may play an important role in determining whether WNV can become established in areas or during seasons when the mosquito densities are too low to provide significant vector-borne transmission [14]. Oral and fecal shedding and/or oral infection have also been reported in some species of mammals and reptiles [10] and references therein.

Very little is currently known about USUV, mainly because it was historically confined to Africa, and because its pathogenicity to humans and animals has only recently been recognised. Moreover, these studies have focused in detecting the virus in dead birds e.g.: [23,28], through serological tests e.g.: [27,31] or through virological or biomolecular testing of blood samples [35]. Fewer studies using oral and cloacal swabs have been carried out to detect USUV [36,37], and only two studies to date have detected the virus [38,39], although in another study it was detected in gastrointestinal tract and kidneys of birds using a biomolecular test [40].

Trentino Alto-Adige, a mountainous Region in northern-eastern Italy, is located on many of the short- and long-distance routes of migratory birds that, from northern Europe, cross the Alps on their way to western Asia or Africa and viceversa [41,42]. So far, only a silent circulation of WNV and USUV in this Region has been detected [14], but the animal species are involved in this cycle have not yet been determined. Despite WNV and USUV sharing some ecological characteristics, knowledge of the natural transmission cycle and of the importance of non-vectorial transmission of USUV are still lacking. Due to the strategic position of this Italian Region in relation to migratory flyways, and the possible role played by migratory birds in the introduction and dispersion of these two *Flaviviruses*, we carried out a biomolecular survey to detect if active virus shedding occurs in migratory birds captured during their seasonal migrations, and to evaluate the role of different species in spreading these viruses.

## Results

A total of 43 birds were captured during the autumn of 2011, 176 during spring 2012, and 103 during autumn 2012 (Table 1). Among the 39 species captured, 18 were long-distance migratory, and 21 short-distance migratory species. Oral and cloacal swabs taken from each individual captured all tested negative for *Flaviviruses*. The positive control tested always positive, and the negative one resulted always negative.

**Table 1:** Bird species tested in Trentino-Alto Adige in 2011 and 2012

**Bird species**		**Family**	**Order**	**Migratory pattern**^**a**^	**2011 Autumn (n)**	**2012 Spring (n)**	**2012 Autumn (n)**	**Total**
**Scientific name**	**Common name**							
*Otus scops*	European scops owl	*Strigidae*	*Strigiformes*	L	-	-	1	1
*Cuculus canorus*	Cuckoo	*Cucilidae*	*Cuculiformes*	L	-	1	-	1
*Jynx torquilla*	Wryneck	*Picidae*	*Piciformes*	L	-	1	-	1
*Aegithalos caudatus*	Long tailed tit	*Egitalidae*	*Passeriformes*	S	-	2	2	4
*Lanius collurio*	Red backed shrike	*Lanidae*	*Passeriformes*	L	-	5	1	6
*Delichon urbica*	House Martin	*Irundinidae*	*Passeriformes*	L	-	-	5	5
*Emberiza schoeniclus*	Reed Bunting	*Emberizidae*	*Passeriformes*	S	-	3	-	3
*Prunella modularis*	Dunnoch	*Prunellidae*	*Passeriformes*	S	2	2	-	4
*Anthus trivialis*	Tree pipit	*Moracillidae*	*Passeriformes*	L	-	-	1	1
*Ficedula hypoleuca*	Pied flycatcher	*Muscicapidae*	*Passeriformes*	S	-	4	6	10
*Muscicapa striata*	Spotted flycatcher	*Muscicapidae*	*Passeriformes*	L	-	12	-	12
*Sylvia borin*	Garden Warbler	*Silvidae*	*Passeriformes*	L	-	9	1	10
*Sylvia curruca*	Lesser Whitethroat	*Silvidae*	*Passeriformes*	S	-	-	3	3
*Hippolais polyglotta*	Melodius Warbler	*Silvidae*	*Passeriformes*	L	-	2	-	2
*Hippolais icterina*	Icterin Warbler	*Silvidae*	*Passeriformes*	L	-	2	-	2
*Acrocephalus scirpaceus*	Reed Warbler	*Silvidae*	*Passeriformes*	L	-	15	-	15
*Acrocephalus palustris*	Marsh Warbler	*Silvidae*	*Passeriformes*	L	-	5	-	5
*Acrocephalus arundinaceus*	Great reed Warbler	*Silvidae*	*Passeriformes*	L	-	6	-	6
*Sylvia atricapilla*	Blackcap	*Silvidae*	*Passeriformes*	S	1	23	1	25
*Locustella naevia*	Grashopper Warbler	*Silvidae*	*Passeriformes*	L	-	1	-	1
*Sylvia melanocephala*	Sardinian Warbler	*Silvidae*	*Passeriformes*	S	-	1	-	1
*Philloscopus trochilus*	Willow Warbler	*Silvidae*	*Passeriformes*	L	-	10	5	15
*Philloscopus collybita*	Chiffchaff	*Silvidae*	*Passeriformes*	S	-	9	-	9
*Philloscopus sibilatrix*	Wood Warbler	*Silvidae*	*Passeriformes*	L	-	2	-	2
*Periparus ater*	Coal Tit	*Paridae*	*Passeriformes*	S	-	-	7	7
*Parus major*	Graet Tit	*Paridae*	*Passeriformes*	S	-	1	-	1
*Phoenicurus phoenicurus*	Redstart	*Turdidae*	*Passeriformes*	L	-	2	2	4
*Phoenicurus ochruros*	Black Redstart	*Turdidae*	*Passeriformes*	S	-	-	3	3
*Oenanthe oenanthe*	Wheatear	*Turdidae*	*Passeriformes*	S	-	-	1	1
*Turdus merula*	Blackbird	*Turdidae*	*Passeriformes*	S	6	6	15	27
*Erithacus rubecula*	Robin	*Turdidae*	*Passeriformes*	S	9	33	24	66
*Turdus viscivorus*	Mistle Thrush	*Turdidae*	*Passeriformes*	S	1	-	-	1
*Turdus philomenos*	Song Thrush	*Turdidae*	*Passeriformes*	S	10	9	18	37
*Turdus iliacus*	Redwing	*Turdidae*	*Passeriformes*	S	-	-	1	1
*Luscinia megarhynchos*	Rufus Nightingale	*Turdidae*	*Passeriformes*	L	-	6	-	6
*Fringilla coelebs*	Chaffinch	*Fringillidae*	*Passeriformes*	S	7	2	3	12
*Coccothraustes coccothraustes*	Hawfinch	*Fringillidae*	*Passeriformes*	S	4	2	-	6
*Carduelis spinus*	Siskin	*Fringillidae*	*Passeriformes*	S	1	-	3	4
*Fringilla montifringilla*	Brambling	*Fringillidae*	*Passeriformes*	S	2	-	-	2
**Total (n)**					**43**	**176**	**103**	**322**

^a^Each species was classified as intrapaleartic (S) and transaharian (L).

## Discussion

The transmission dynamics of *Flaviviruses* are based on a complex relationship among virus occurrence, host and vector species community composition, host behaviour, vector host preferences and competence, and environmental and climatic factors, making each spillover event a unique phenomenon resulting from the combination of all these factors [34] and references therein.

Since oro-fecal shedding is an important amplification route for these viruses, assessing the rate of oro-fecal shedding in various species is important to identify the amplification chain [7,8,10,11,34] and references therein. Bird species differ in their susceptibility to WNV and USUV infection. For example, *Passeriformes* and *Strigiformes* are highly susceptible to USUV infection e.g.: [23,26,28,35] and *Passeriformes*, *Charadriiformes* and *Strigiformes* are the principal host reservoirs and amplificators of WNV, due to their long-lasting and high levels of viremia e.g.: [34,43,44]. Moreover, it has been suggested that a single species can act as a super-spreader of WNV [6].

In previous studies, the oro-fecal shedding of USUV was detected in domestic goose (*Anser anser f. domestica*[38]) and domestic chicken (*Gallus domesticus*[39]). Alternatively, 14 out of a total of 39 bird species analysed (for e.g.: Greenfinch *Carduelis chloris*, Great Tit *Parus major*, Pied Flycatcher *Ficedula hypoleuca*, Willow Warbler *Phylloscopus trochilus*, Icterine Warbler *Hippolais icterina*, Blackcap *Sylvia atricapilla*, Blackbird, European Robin *Erithacus rubecula*) previously tested negative in study also carried out in Italy [36]. Moreover, shedding was also not evident in the Eurasian Jay (*Garrulus glandarius*), domestic chicken, European Nightjar (*Caprimulgus europaeus*), European Bee-eater (*Merops apiaster*), Barn Swallow, Cetti’s Warbler (*Cettia cetti*), Blue Tit (*Parus ceruleus*) [36], and for 11 species belonging to the order *Anseriformes* tested in Finland [37].

With respect to WNV, the species tested by [36], the 11 species belonging to the order *Anseriformes*, screened by [37], and the individuals belonging to the family *Corvidae* of British Colombia tested by [45] resulted negative for WNV shedding. In India, 119 species belonging to 30 families and in particular *Cuculidae*, *Motacillidae*, *Silvidae*, *Turdidae* (order *Passeriformes*) and *Strigidae* (order *Strigiformes*) were analysed and all tested negative [46]. This further corroborates the results of the current study. A study carried out in Spain did not find oral shedding in species belonging to several families, namely *Threskiornithidae* and *Accipitridae*[31]. The tracheal and cloacal swabs tested in Germany were negative [47]. The tested birds belonged to order *Charadriiformes* (e.g.: Ringed Plover *Charadrius hiaticula*, Little ringed Plover *Charadrius dubius*, Black-headed Gull *Larus ridibundus*), some to genus *Calidris* and *Tringa*, some to the orders *Anseriformes* (*Anas* spp.), *Gruiformes* (Water Rail *Rallus aquaticus*, Eurasian Coot *Fulica atra*) and *Passeriformes*, family *Motacillidae* (for e.g.: White Wagtail *Motacilla alba*, Meadow Pipit *Anthus pratensis*) and others to the family *Corvidae* (Hooded Crow *Corvus corone cornix*).

On the other hand, additional studies have detected oro-fecal shedding of WNV in bird species of different families and orders. These include *Corvidae,* such as American Crows (*Corvus brachyrhynchos*), Fish Crows (*Corvus ossifragus*), Blue Jays (*Cyanocitta cristata*), Common Ravens (*Corvus corax*), Black-billed Magpies (*Pica pica*), Little Raven (*Corvus mellori*) for e.g.: [11,48-52]; *Anatidae* (order *Anseriformes*) such as Canada Goose (*Branta canadensis*), Wild Mallard (*Anas platyrhynchos*) and Domestic Goose [11,20,53]; *Galliformes*, such as Northern Bobwhite (*Colinus virginianus*), Turkey (*Meleagridis gallopavo*), domestic chicken, Red-legged Partridge (*Alectoris rufa*) [11,54,55] and references therein; *Gruiformes*, such as American Coot (*Fulica americana*) [11]; *Charadriiformes*, such us: Killdeer (*Charadrius vociferus*), Ring-billed Gull (*Larus delawarensis*) [49]; *Columbiformes*, such as Mourning Dove (*Zenaida macroura*) and Rock Dove (*Columba livia*) [11]; *Psittaciformes*, such as Monk Parakeet (*Myiopsitta monachus*) and Budgerigar (*Melopsittacus undulatus*) [11]; *Passeriformes*, such as American Robin (*Turdus migratorius*), Common Grackle (*Quiscalus quiscula*), House Finch (*Carpodacus mexicanus*), House Sparrow (*Passer domesticus*), Great-tailed Grackles (*Quiscalus mexicanus*), Cedar Waxwing (*Bombycilla cedrorum*), Northern Mockingbird (*Mimus polyglottus*), Barn Swallow (*Hirundo rustica*), Cliff Swallow (*Petrochelidon pyrrhonota*) [49,56,57]; several species of diurnal and nocturnal raptors, such as Swainson’s Hawk (*Buteo swainsoni*), Ferruginous Hawk (*Buteo regalis*), Peregrine Falcon (*Falco peregrinus*), Golden Eagle (*Aquila chrysaetos*), American Kestrel (*Falco sparverius*), and some species of North American owls (family *Strigidae*) like Great Horned Owl, (*Bubo virginianus*) [11,49,58,59].

The results of our study further corroborate the results of a previous study also carried out in Italy, which found there was no evident oro-faecal shedding of USUV in the families *Fringillidae*, *Lanidae*, *Paridae*, *Muscicapidae*, *Silvidae*, *Turdidae*, *Hirundinidae* and *Picidae*[36]. Our results also seem to suggest that birds belonging to the families *Motacillidae*, *Prunellidae*, *Emberizidae*, *Cuculidae*, *Egitalidae*, *Strigidae*, previously never screened for USUV, may not be important shedders of this virus.

Considering the migratory birds tested in Italy so far, what has been said for USUV is also valid for WNV. Moreover, this has also been confirmed in India in birds belonging to the families *Cuculidae*, *Motacillidae*, *Silvidae*, *Turdidae* (order *Passeriformes*) and *Strigidae* (order *Strigiformes*) [46] and in Germany for *Motacillidae*[47]. Of the studies that found oro-fecal shedding for WNV, only one was carried out in Europe, but is not possible to compare it with our research mainly for two reasons: firstly, it studied a species belonging to the order *Galliformes* that was not included in the current study; and secondly, the birds were experimentally infected with the virus, and so the results may not reflect those seen in natural conditions in the wild [54]. The other studies were carried out in America and Australia and principally focused on taxonomic groups that are different from the ones that were included in our research (orders: *Columbiformes*, *Psittaciformes*, *Charadriiformes*, *Gruiformes*, *Galliformes*, *Anseriformes*, *Falconiformes*). Studies that have been carried out on *Strigiformes* and *Passeriformes*, also investigated different species to the ones included in our study (for e.g.: family *Corvidae*, American Robin, Common Grackle, House Finch, House Sparrow, Cliff Swallow, Golden Eagle, *Bubo* spp., *Buteo* spp., *Falco* spp.).

Accordingly, it seems that the oro-fecal shedding of USUV and WNV in *Cuculiformes* and *Piciformes* is not intense or it lasts only few days. Regarding *Strigiformes* and *Passeriformes*, their shedding seems low also for USUV, but for WNV, various families or species could have an important role, such as *Corvidae*, *Hirundinidae*, *Icteridae*, *Turdidae*, *Fringillidae*, *Passeridae*, *Bombycillidae*, *Mimidae* and *Tytonidae*. There are several factors that could explain these different results, for example, the limited number of subjects that were tested and the taxonomical differences between the birds screened. Also, an additional reason could be the period of the year during which the study was carried out in relation to the bird’s physiology: migration requires morphological and physiological changes [33] that could interfere with the viral replication. Moreover, the oro-fecal shedding generally lasts less than 10 days [11], thus being not easy to detect in clinically healthy animals as in those individuals who are migrating. Besides, the shedding is not always followed by virus transmission e.g.: [10,54] and references therein.

**Figure 1:**
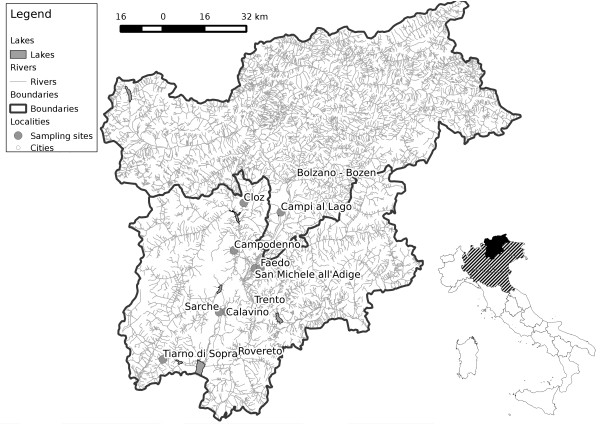
**Bird sampling sites.** Italian map insert: black area, sampling region of Trentino-Alto Adige; hatched area, neighbouring regions (Veneto, Lombardy, Emilia Romagna, Friuli-Venezia Giulia) with active WNV and USUV circulation.

Taking into account the need to identify the species and the timing of WNV and USUV amplification, the absence of active shedding detected in this study may also justify the absence of clinically reportable cases of spillovers events to human and animal in Trentino-Alto Adige. Furthermore, at present, no human or animal clinical case of diseases or infections caused by *Flaviviruses* have been recorded in this region of Italy. Their circulation is then apparently very limited, in contrast to the high number of cases and the pathogenicity observed in animals, mosquitoes and humans in the neighbouring regions (Veneto, Lombardy, Emilia-Romagna, Friuli-Venezia Giulia: Figure 1). A possible explanation of this observed epidemiological pattern could be due to the low density of mosquitoes observed in this area as a result of a low habitat suitability for *Culex* spp.: a combination of low anthropization and mountainous orography of the territory, of which about 78% lies over 1,000 m above sea level, and about 55% is covered by coniferous and deciduous forests, with a temperate-oceanic climate, although a sub-Mediteranean climate can be found near Lake Garda. It is not the case that most of the detections of *Flaviviruses* monitored in this region were obtained in the region around Lake Garda, which provides a suitable habitat for many species of mosquitoes, including *Cx. pipiens* and *Aedes albopictus*[60,61], Rizzoli A: personal communication. This is consistent with the observation that viruses transmitted by mosquitoes are more frequently linked to mild climate, irrigated areas, wetlands and marshes with abundant mosquito and bird populations, especially migratory birds for e.g.: [16,46,47,62]. Another co-factor to be considered is the presence of a high avian biodiversity observed in the region compared to other neighbouring regions. The relationships among high host diversity and low virus spillover have been observed in several disease models, including WNV [6,63-65].

## Conclusions

In this study we did not identify active oro-fecal shedding of WNV and USUV in 322 individual birds belonging to 18 transaharian and 21 intrapaleartic species. The lack of detection of active virus shedding in these species, however, does not exclude the circulation of these viruses within the Region of Trentino-Alto Adige, as noted in a previous study [14]. Considering the high rate of animals and goods movements into this territory, and possible future climatic changes, the temporal and spatial dynamics of pathogens, vectors and avian hosts could also change [66]; therefore, the circulation of *Flaviviruses* in Trentino Alto-Adige needs to be carefully monitored in the future.

## Methods

### Bird netting

Sample collection was carried out in Trentino-Alto Adige region during ringing campaigns in autumn 2011 and 2012 (September and October) and spring 2012 (March to May). Intrapaleartic and transaharian migratory birds were captured by ornithologists using net labyrinths authorized by ISPRA (Istituto Superiore per la Protezione e la Ricerca Ambientale, Ozzano dell’Emilia, Bologna, Italy) within the European Union for Bird Ringing (EURING) which includes ethical approval. The research protocol was also approved by the Wildlife Management Committee of the Autonomous Province of Trento (Italy). These activities are carried out to provide data on migration patterns, demography and ecological processes. The sampling sites included: Faedo (Trento) and Tiarno di Sopra (Trento) during the 2011 autumnal ringing campaing; Cloz (Trento), Campi al lago (Caldaro, Bolzano), Campodenno (Trento), Calavino (Trento) and Sarche (Trento) during the 2012 spring ringing campaing; Faedo (Trento), Tiarno di Sopra (Trento) and San Michele all’Adige (Trento) during the 2012 autumnal ringing campaing (Figure 1).

### Sampling

Oral and cloacal samples were taken from each captured bird using sterile swabs with transport medium AMIES without charcoal, in polypropylene tubes Ø 12×150 mm (Nuova Aptaca S.r.l., Canelli - AT, Italy). Samples were kept refrigerated during transport to the laboratory, where they were stored at -80°C until analysis. Each bird was manipulated only for few minutes and prior to its release, each one was marked by standard procedures using metal leg rings, according to EURING procedures. Date of capture, species, ring number, age, weight and other morphobiometric parameters were recorded for each individual.

### RNA extraction and Polymerase Chain Reactions (PCRs)

Molecular analyses were performed in the laboratory of Veterinary Sciences Department of University of Torino (Grugliasco, Torino - Italy). For RNA extraction, each swab was dissolved in 200 μl of phosphate-buffered saline (PBS) buffer (Sigma-Aldrich, Milan, Italy) and the suspension obtained was centrifuged for 5 minute at 8000 rpm. 140 μl of the supernatant was added to 560 μl of Buffer AVL and carrier RNA, prepared according to QIAamp® Viral RNA Mini Handbook (Qiagen, Hilden, Germany). The samples were then processed following this protocol. In the final step, RNA was eluted in 60 μl of Buffer AVE. After quantification with Thermo Scientific Nanodrop 2000 (Thermo-Scientific, Euroclone, Milan, Italy), up to 1 μg of RNA was reverse-transcribed according to QiagenQuantiTect® Reverse Transcription Kit Handbook (Qiagen, Hilden, Germany). For the screening of *Flaviviruses*, we used a generic nested RT-PCR that amplifies a region of the NS5 gene that is well-conserved within this genus, according to [67], with modifications (using a volume of 5 μl of the cDNA of the first PCR, 5 U of HotStarTaq DNA Polymerase (Qiagen, Hilden, Germany), 40 pmol of each generic Flavivirus primer (Flavi1+, Flavi1-), and 10 nmol of each dNTP). In the nested PCR mix, 1 μl of PCR product from the first reaction was added to 49 μl of reaction mix composed by 1.25 U of HotStarTaq DNA Polymerase, 40 pmol of each primer (Flavi2+, Flavi2-), and 10 nmol for each dNTP. Finally, the products of the nested PCR were analysed by electrophoresis with a 1.5% (w/v%) agarose gel (Sigma-Aldrich, Milan, Italy) and visualized by staining with 0.1% (w/v%) of ethidium bromide. Positive and negative controls were included in the analyses.

## Abbreviations

EURING: European Union for Bird Ringing; ISPRA: Istituto Superiore per la Protezione e la Ricerca Ambientale; PBS-buffer: Phosphate-buffered saline; PCRs: Polymerase chain reactions; USUV: Usutu virus; WNV: West Nile virus.

## Competing interests

The authors declare that they have no competing interests.

## Authors’ contributions

MG and AR conceived and directed the study. MG, DA and FR carried out the bird netting and swabs collection. MG carried out the molecular analysis and drafted the manuscript. LB participated in the molecular analysis. MaG was involved in the interpretation of the data. All authors participated in the revision of the manuscript and approve the submitted version. All authors read and approved the final manuscript.
